# The role of the oxytocin system in the resilience of patients with breast cancer

**DOI:** 10.3389/fonc.2023.1187477

**Published:** 2023-09-13

**Authors:** Shaochun Liu, Runze Huang, Anlong Li, Sheng Yu, Senbang Yao, Jian Xu, Lingxue Tang, Wen Li, Chen Gan, Huaidong Cheng

**Affiliations:** ^1^ Department of Oncology, The Second Hospital of Anhui Medical University, Hefei, Anhui, China; ^2^ Shenzhen Clinical Medical School of Southern Medical University, Guangzhou, China; ^3^ Department of Oncology, Shenzhen Hospital of Southern Medical University, Shenzhen, Guangdong, China

**Keywords:** breast cancer, resilience, oxytocin system, behavioral interventions, psychotherapy

## Abstract

Breast cancer is a grave traumatic experience that can profoundly compromise patients’ psychological resilience, impacting their overall quality of life. The oxytocin system represents one of the essential neurobiological bases of psychological resilience and plays a critical role in regulating resilience in response to social or traumatic events during adulthood. Oxytocin, through its direct interaction with peripheral or central oxytocin receptors, has been found to have a significant impact on regulating social behavior. However, the precise mechanism by which the activation of peripheral oxytocin receptors leads to improved social is still not completely comprehended and requires additional research. Its activation can modulate psychological resilience by influencing estrogen and its receptors, the hypothalamic-pituitary-adrenal axis, thyroid function, 5-hydroxytryptamine metabolism levels, and arginine pressure release in breast cancer patients. Various interventions, including psychotherapy and behavioral measures, have been employed to improve the psychological resilience of breast cancer patients. The potential effectiveness of such interventions may be underpinned by their ability to modulate oxytocin release levels. This review provides an overview of the oxytocin system and resilience in breast cancer patients and identifies possible future research directions and interventions.

## 1 Introduction

### 1.1 Development of the concept of resilience

In 1974, Anthony introduced the concept of psychological resilience based on child psychological development, which arises from successful adaptation to adversity (1). Masten et al. argue that this concept persists throughout life and is a dynamic psychological process that constantly adjusts to the internal and external environment (2); similarly, Bonanno et al. suggest that resilience represents a stable trajectory for individuals to maintain healthy functioning after experiencing highly adverse events (3). In 2014, Southwick et al. summarized that resilience should be defined according to the individual’s stage and environment and the types of traumatic events encountered (4). Continued advancements in life sciences and biomedical engineering have provided researchers with additional means to investigate the biological processes that shape and develop this concept (5). It has been demonstrated that psychological resilience is closely linked to rehabilitating malignant tumors and chronic diseases and that good psychological resilience can prevent disease onset and effectively maintain a sense of well-being in life (6). Thus, these findings have shown that good psychological resilience protects against diseases and supports overall well-being.

### 1.2 Involvement of the oxytocin system in shaping resilience

In 2020, Feldman and colleagues proposed a neurobiological model of resilience based on the oxytocin system, the affiliative brain, and biobehavioral synchrony from an evolutionary and sociological perspective (7). This model identifies plasticity, sociality, and meaning as the three key features of resilience, which provide theoretical possibilities for improving resilience. The oxytocin system, one of the three bases of resilience, is interconnected with the affiliative brain and biobehavioral synchrony and is involved in shaping psychological resilience. Oxytocin, as the endogenous core substance of this system, is a multi-potent peptide hormone with unique chemical properties that can act as an anti-inflammatory and antioxidant molecule in response to stress caused by adversity and trauma (8, 9). Oxytocin receptors belong to the group of seven transmembrane G-protein-coupled receptors, consisting of 389 amino acid residues and belonging to the class I G protein-coupled receptor family (10); the distribution of oxytocin receptors in the brain may be the histological basis for the involvement of the oxytocin system in shaping psychological resilience. These properties may help explain the benefits of positive social experiences and have drawn attention to this system as a possible treatment for various disorders. Of particular interest, the oxytocin system regulates resilience throughout a woman’s life, from brain maturation in the mother’s womb through pregnancy, childbirth, breastfeeding, and various social behaviors and connections (11).

### 1.3 Distribution and expression of central and peripheral oxytocin receptors

Oxytocin is primarily synthesized in the paraventricular nucleus (PVN) and supraoptic nucleus (SON) of the hypothalamus, specifically in large and small cell neurons (12). Magnocellular oxytocin neurons release oxytocin into the peripheral blood through the posterior pituitary gland. These neurons also have significant central projections that innervate nerves in the forebrain, contributing to regulating various behaviors. On the other hand, parvocellular neurons, which are smaller in size, mainly project to the posterior brain and spinal cord. They are thought to regulate functions like cardiovascular function, breathing, feeding behavior, and nociception (13).

Furthermore, studies have indicated that Magnocellular oxytocin neurons may incidentally project to more than 50 different brain regions, including the caudate Putnam (14). This extensive investigation suggests that these neurons play a crucial role in central activities mediated by oxytocin, such as fear attenuation, social interaction, and movement. In summary, the oxytocin system is a complex network of peripheral and central activity regulated by diverse neuronal populations and pathways, serving a wide range of physiological and behavioral functions.

In animal experiments, oxytocin injection into different brain regions produced other physiological or social behavioral effects. In primate experiments, blood pressure decreased with intracerebroventricular oxytocin administration (15). Social interactions can promote oxytocin production, and oxytocin can promote bonding or attachment between individuals (e.g., mother-infant and sexual partners) (16). Oxytocin may also have other effects (17). For example, naloxone antagonizes the persistent effects of oxytocin in the tail-flick test, suggesting that oxytocin may increase the activity of endogenous opioids (18). Oxytocin receptor gene-negative mice have a lower proportion of hippocampal neurons expressing GABAergic synapses, an imbalance in glutamate-GABA transmission, and an upregulated number of V1a receptors in the hippocampus, showing abnormal social skills, impaired cognitive flexibility (associated with hippocampal function) and reduced distress at separation from the mother. Oxytocin restores symptoms such as cognitive flexibility and seizure susceptibility in mice (19). Injecting oxytocin into the hippocampus or intraperitoneally in rats stimulates cell proliferation in the dentate gyrus of the hippocampus. The oxytocin-induced increase in newborn neurons in the DG may help reduce anxiety and enhance learning ability (20).

The distribution of oxytocin receptors in the brain also affects psychological resilience. It has been shown that the expression of distributed oxytocin receptors in the ACC (anterior cingulate area) regulates anxious behavior. Microinjection of oxytocin in mice’s ACC significantly increased the threshold of mechanical foot contraction response and reduced chronic pain-induced anxiety in neurologically injured mice by selectively blocking the maintenance of presynaptic long-duration enhancement (21). In clinical studies, transnasal administration of oxytocin to healthy female youths activated the ACC and attenuated the neurological effects of subthreshold threat stimuli, acting as an anxiolytic (22). In addition, transnasal administration of oxytocin has been used in randomized controlled trials in various neuropsychiatric disorders, such as Autism Spectrum Disorder, Generalized/Social Anxiety Disorder, and Posttraumatic Stress Disorder; some of these studies are listed in **Table 1**. In most studies, transnasal administration of oxytocin produced beneficial effects for the patients. However, some studies did not find significant efficacy with intranasal oxytocin administration. For example, the benefit of oxytocin for social functioning in patients with autism spectrum disorders was not superior to the placebo (25). Thus, in breast cancer patients, changes in plasma oxytocin levels and peripheral oxytocin receptor distribution may influence psychological resilience and the ability to mentally cope with a crisis or quickly return to pre-crisis status. However, it’s important to note that the central effects of oxytocin, although potentially significant, are less understood due to limited studies on primary oxytocin levels and receptor distribution. This area could be a valuable avenue for future investigation, even though intranasal oxytocin does not always function as expected.

**Table 1:** Use of intranasal oxytocin in selected neuropsychiatric disorders.

Types of disease	Source of drugs	Dose of the drug administered	Time of administration	Sample Size	Subjects	Effect	Reference
ASD	Tergus Pharma	4-8IU/day	Once daily for 24 weeks	355	3 to 17 patients with ASD children and adolescents	Social or cognitive functioning—	(23)
	Novartis, Switzerland	48IU per dose	Once a day for 6 weeks	106	People aged 18-48 years with autistic disorder, Asperger’s disorder or pervasive developmental disorder	Autism Diagnostic Observation Schedule (ADOS) reciprocity↓	(24)
	F.Hoffmann-La Roche Ltd, Basel, Switzerland	10-mg balovaptan adult-equivalent dose per dose	Once daily for 24 weeks	599	Between the ages of 5 and 17 years, patients diagnosed with ASD	The efficacy of social interaction and communication in the population —	(25)
	–	18 or 24 IU per dose	The treatment was given once daily for 8 weeks	16	Young men aged 12 to 19 years diagnosed with autism or Asperger’s syndrome	Emotion recognition ability ↑	(26)
	Farma Holding, Oslo, Norway	8 or 24 IU per dose	Treatment 3 times, each time intervals of 1 ~ 72 hours	17	Adult men with ASD	emotion salience↑	(27)
GSAD	··	24 IU per dose	Two treatments, The interval between each treatment was 1 week	36	Male patients with GSAD aged 19 to 55 years	The severity of social anxiety ↓ Integration and regulation of social responses ↑ Amygdala response to fear ↓	(28–30)
PTSD	Defiante Farmaceutica, S.A., Funchal Portugal	40 IU per dose	1 treatment	40	Police officers diagnosed with PTSD	sensitivity for social support and therapeutic alliance↑↑	(31)
	Defiante Farmaceutica, Funchal, Portugal	40 IU per dose	Twice a day for 8 days	1107	Adults with more than moderate acute distress	The severity of acute PTSD symptoms ↓	(32)
	Novartis, Brazil	24 IU per dose	It was received twice in a week	35	Adult female patients with PTSD	the intensity of provoked PTSD symptoms↓↓	(33)
SZ	Novartis, Basel Switzerland	40 IU per dose	Once daily for 1 week and twice daily after that	20	Patients with SCID-confirmed DSM-IV diagnosis of schizophrenia	verbal memory↑↑ scores on the Positive and Negative Symptom Scale↓	(34, 35)
	Novartis	24IU per dose	The treatment was given twice daily for 14 days	23	Patients 18 to 55 years of age with a DSM-IV diagnosis of paranoid or undifferentiated schizophrenia	Social Cognitive deficits ↓	(36)
	Seoul National University Hospital	40IU per dose	The drugs were administered twice at an interval of one week	32	Male patients with schizophrenia	activity for happy faces↑	(37)
FTD	Novartis	24IU per dose	The drug was administered once	20	Patients who met the consensus criteria for FTD	Patients who met the consensus criteria for FTD ↑	(38)
	Novartis, Bern, Switzerland	24IU per session	Three doses were administered every 10 minutes	51	Patients who met the consensus criteria for FTD	Activity in limbic regions associated with the processing of emotional expressions ↑	(39)

ASD, Autism Spectrum Disorder; GSAD, Generalized/Social Anxiety Disorder; PTSD, Post-traumatic Stress Disorder; SZ, Schizophrenia; FTD, Frontotemporal Dementia; DSM, The Diagnostic and Statistical Manual of Mental Disorders.

Based on a review of the literature, the effects are listed as follows in clinical randomized controlled trials of different designs: ↑= improved; ↓ = reduced; —= no significant change.

### 1.4 Low level of resilience in breast cancer patients

Breast cancer is the most commonly diagnosed cancer among women, accounting for 11.7% of all cases, and one in six women with cancer dies from breast cancer (40). Breast cancer presents a clinical challenge with up to ten different molecular subtypes, including ductal A, ductal B, her2-enriched, and basal-like. Insulin-regulated aminopeptidase (IRAP), the only enzyme that cleaves oxytocin, is correlated with circulating levels of oxytocin and may affect mammary breast tumor tissue metabolism by modulating GLUT4 and angiotensin II (ATII) levels (41–43). In the context of breast cancer treatment, the American Society of Clinical Oncology (ASCO) guidelines emphasize using neoadjuvant systemic therapy, including chemotherapy, endocrine therapy, and targeted therapy, to improve outcomes in patients with invasive breast cancer (44). Diagnosis, symptoms, treatment, surgery, and the impact of breast cancer on patients’ lives can all induce significant stress, leading to higher rates of anxiety and depression in breast cancer patients compared to non-cancerous women (45). These factors contribute to the lower level of resilience commonly observed in breast cancer patients.

### 1.5 Relationship between changes in the oxytocin system and resilience regulation in breast cancer patients

In breast cancer patients, alterations in the oxytocin system are associated with the regulation of resilience. The development of breast cancer significantly impacts the oxytocin system, and studies have shown that the level of resilience in these patients may be related to the severity of anxiety or depressive symptoms (46). Interestingly, not all women with breast cancer experience severe psychological distress and those with higher levels of resilience exhibit lower probabilities or degrees of such symptoms (47). Resilience in breast cancer patients is both a state and a trait, as demonstrated by a study investigating resilience’s role in these patients (48). In the context of the COVID-19 pandemic, it was found that breast cancer patients with higher levels of resilience had fewer concerns about tumor progression due to COVID-19 infection (49). Resilience, therefore, plays a crucial role in the quality of life of breast cancer patients and their ability to cope with diagnosis, treatment, and recovery.

Notably, the level of psychological resilience in breast cancer patients is influenced by various factors such as disease stage, treatment duration, social support, and level of education. Patients with early clinical stages, short treatment courses, high social support, and education tend to have higher resilience (50–52). Conversely, more treatment courses and faster disease progression are associated with lower resilience (53). Improving the resilience of breast cancer patients is crucial for enhancing their quality of life, independent of their survival. To enhance resilience, non-pharmacological interventions based on behavioral and sociological theories have been explored, including spirituality, supportive-expressive group therapy, art and movement therapies, nursing interventions, and educational components (54–56). Interestingly, a study of depressed patients using psychodynamics demonstrated that the more significant the change in oxytocin response during treatment, the more influential the improvement in depressive symptoms (57), similar to another study suggesting that resilience may be a preventive factor for depression (58). Therefore, it is plausible to hypothesize that changes in the oxytocin system are one of the pathways through which resilience is modulated in breast cancer patients.

### 1.6 Methodology and purpose

This article aims to analyze the interaction of the oxytocin system with social, genetic, physiological, and pathological factors in breast cancer resilience and propose a theoretical regulatory model. We conducted a systematic literature search from January 1990 to May 2023 using specific MESH terms and key terms. The data source was PubMed. The search terms were ((“oxytocin”[MeSH Terms] OR “oxytocin”[All Fields]) OR (“breast neoplasms”[MeSH Terms] OR (“breast”[All Fields] AND) “neoplasms”[All Fields]) OR “breast neoplasms”[All Fields] OR (“breast”[All Fields] AND “cancer”[All Fields]) OR “breast cancer”[All Fields])) AND (“resilience, psychological”[MeSH Terms] OR (“resilience”[All Fields] AND “psychological”[All Fields]) OR “psychological resilience”[All Fields] OR “resilience”[All Fields]). We focused on original research articles and reviews in English and excluded non-English studies.

## 2 The oxytocin system and the mammary gland

### 2.1 The impact of oxytocin on mammary tissue during pregnancy and lactation

The oxytocin system has been demonstrated to affect women’s mammary tissue during pregnancy or lactation (41, 59, 60). Medications administered during pregnancy or delivery may impact oxytocin secretion, leading to impaired milk production or delayed lactation initiation (61). However, there is a paucity of research on the non-lactating period (46). Several studies have reported that stimuli such as mechanical pumps and tactile sensations can promote elevated plasma oxytocin levels (62). The activated noradrenergic, histaminergic, and glutamatergic receptor systems may stimulate central oxytocin release during lactation. For example, norepinephrine has been shown to stimulate central oxytocin receptors during gestation (63). Breastfeeding mothers have been observed to exhibit higher plasma and salivary oxytocin levels (64).

### 2.2 Oxytocin’s protective role against breast cancer development

The predominant viewpoint suggests that oxytocin functions as a preventive factor for breast cancer by acting on myoepithelial cells, which relieves the expansion of secretory vesicles and facilitates the elimination of carcinogenic substances, ultimately decreasing the risk of breast tumor development (65, 66). Downregulation of oxytocin-related genes FOS, ITPR1, RCAN1, CAMK2D, and CACNA2D was observed in breast cancer samples, implying a correlation between the expression of this endogenous molecule and breast tumor malignancy (67). Additionally, oxytocin inhibited estrogen-induced cell growth and enhanced tamoxifen’s inhibitory effect on cell proliferation (10). Intranasal administration of oxytocin has been proposed as a breast cancer prevention strategy, where nipple fluid samples collected for testing by intranasal Oxytocin injection can be utilized as a screening tool for individuals at high risk of breast cancer (68).

### 2.3 Oxytocin receptors in breast cancer: implications for estrogen receptors and metastasis

While oxytocin receptors are expressed in various human breast cancer cell lines, their significance in developing and diagnosing breast cancer remains unclear. Some researchers have proposed that the distribution of oxytocin receptors in breast tumor tissue correlates with the expression of estrogen receptors (ER) (46, 69, 70). In a study on triple-negative breast cancer tissues, researchers found that MDA-MB-231 cells overexpressing oxytocin receptors were more sensitive to Epidermal Growth Factor (EGF) and demonstrated enhanced migration. The study suggested that high oxytocin receptor levels are associated with increased EGF sensitivity and that oxytocin receptors promote EGF-stimulated RSK activation via the mTOR pathway, leading to downstream rpS6 activation and enhanced migration of breast cancer cells. The researchers also found that rapamycin, a selective inhibitor of mTOR, reduced the migration of oxytocin receptor overexpressing cells (71). However, this study was only conducted on cellular models; additional clinical or animal studies have yet to support this finding. Contrastingly, one study found higher oxytocin receptor levels in healthy breast tissue compared to cancerous tissue. This suggests that reduced oxytocin receptor expression in breast tissue may promote carcinogenesis (72). This study did not investigate triple-negative breast cancer tissue, so it could not refute the earlier study’s findings. Although oxytocin receptor signaling may stimulate some aspects of breast cancer progression *in vitro*, oxytocin’s net impact *in vivo* seems to inhibit cancer, hinting at complex context-dependent effects. Further research must elucidate how oxytocin signaling differentially affects healthy versus malignant breast tissue through changes in oxytocin receptor levels, downstream pathways, and interacting factors.

## 3 The oxytocin system and resilience

### 3.1 The role of oxytocin system in maternal depression and neonatal resilience formation

Maternal depression can negatively affect children’s mental health, increasing the risk of developing mental illness later in life (73, 74). The oxytocin system may be involved in the transmission of depression from mother to child and the establishment of neonatal resilience. During the early sensitive period of maternal care, the infant’s oxytocin system is formed and highly influenced by epigenetics. The intergenerational transmission of the oxytocin system suggests that maternal oxytocin levels may influence maternal care and shape the infant’s oxytocin system (74, 75). Social synchronization, the mutual adaptation between parent and child, is one of the bases of resilience formation. A long-term follow-up study found that when a mother experiences depression, high salivary oxytocin levels in the child suggest higher synchronization and a lower likelihood of developing mood or psychiatric disorders, indicating resilience (74).

### 3.2 Oxytocin receptors as predictors of psychological resilience: implications for breast cancer patients

Oxytocin receptors have been linked to differences in personal psychological resources such as self-esteem, optimism, and mastery, considered protective factors for developing resilient functioning. Furthermore, attachment and relationship quality, critical components of resilient functioning, have also been shown to be associated with oxytocin receptors. For instance, non-maltreated children with AA or AG genotypes of the oxytocin receptor have been found to exhibit higher levels of psychological resilience compared to maltreated children with identical genotypes (76). The impact of the oxytocin system on cognitive and social functioning has been widely discussed, although discrepancies in findings across studies have been observed. This may be related to variations in oxytocin receptor genotypes. The effect of oxytocin receptor variants on social functioning in individuals is mainly derived from two single nucleotide polymorphisms located in the third intron, rs53576 and rs2254298 (77). The association between childhood family environment and psychological resilience varies depending on the oxytocin receptor rs53576 genotype, with a stronger correlation observed in individuals with the AG genotype (78). In a healthy Korean population, psychological resilience scores were strongly correlated with the oxytocin receptor SNP rs53576 genotype, with GG carriers exhibiting the highest level of resilience and each additional copy of the A allele resulting in a 3.84 decrease in CD-RISC score (79).

Conversely, veterans with insecure attachment and the A allele of the oxytocin receptor SNP rs53576 were found to be at a higher risk of PTSD (76). The diverse roles of psychological resilience in different domains have been proposed, with some studies suggesting that oxytocin receptor DNA methylation in children can predict (80). Hence, the oxytocin receptor genotype holds promise as a predictor of resilience in breast cancer patients.

### 3.3 Insights from prairie vole model: unraveling the oxytocin system’s influence on psychological resilience and social behavior

The prairie vole model with standard monogamy is highly informative in revealing individual differences in the effects and mechanisms of the oxytocin system on psychological resilience (81). Notably, studies have demonstrated the crucial role of the oxytocin system in regulating the social behavior of steppe voles. Prairie voles deficient in the oxytocin receptor gene exhibit reduced empathic responses and helpful behavior compared to controls (82). Numerous studies have investigated the pathways by which the oxytocin system is involved in the social behavior of steppe voles. For instance, in neonatally isolated female prairie voles, the distribution of oxytocin receptors in the nucleus ambiguous (NAcc) is significantly correlated with partner preference (PP) behavior, and those females with high oxytocin receptor densities in the NAcc demonstrate resilience to the effects of neonatal social isolation on later PP behavior. Oxytocin activity in the NAcc may mediate the response to adverse life events, vulnerability, or toughness (83). In contrast, early social deprivation in prairie voles impairs the formation of social connections and increases anxiety in adulthood, while touch stimulation restores some of these functions (increasing EGR-1 gene immunoreactivity in hypothalamic oxytocin neurons and inducing oxytocin signaling).

Furthermore, using the MC3/4R (Melanocortin 3/4 Receptors) agonist MTII has been found to activate oxytocin neurons and enhance stimulus-induced oxytocin release in the brains of adult prairie voles, which could potentially mitigate the negative impact of isolation on adult relationships. In particular, MTII has been shown to stimulate EGR-1 immunoreactivity in oxytocin neurons and increase hypertonic saline-induced oxytocin release to the NAcc (65). In contrast, the administration of oxytocin A, an oxytocin receptor antagonist, to male voles has been found to increase the binding of Arginine vasopressin (AVP) pressor to V1aR in the ventral pallidum, a region where dopamine also binds. This finding suggests that oxytocin may affect dopamine metabolic processes, potentially explaining its association with negative coping behaviors (84).

## 4 Pathways involved in the regulation of resilience in breast cancer patients by the oxytocin system

Oxytocin is a neuropeptide that plays a crucial role in regulating mood, behavior, and cardiovascular function by increasing serotonin activity in the brain (85). It also interacts with other body systems, such as the *AVP* system, which is responsible for cardiovascular regulation, and the thyroid system, which is required for metabolism and mood (86, 87). Due to changes in estrogen levels, breast cancer patients may experience decreased oxytocin activity, leading to thyroid dysfunction, decreased psychological tolerance, and symptoms such as cognitive changes, fatigue, and appetite disturbances (88). Breast cancer cells may also aberrantly express *AVP*, affecting cardiovascular function and emotional behavior (89). Because these systems are interdependent, changes in one system can significantly impact the others. Understanding these interactions is critical for effective treatment and improving the quality of life of breast cancer patients.

### 4.1 Regulation of resilience in breast cancer patients by influencing psychological and physiological phenomena

Oxytocin facilitates social behavior and adaptive responses to threats by activating the vagus nerve and reducing fear and immobility, while abnormal oxytocin levels in trauma can lead to dissociation and impaired health. Oxytocin regulates social and emotional responses through effects on the vagus nerve, enabling adaptive coping in stress, while dysfunction may contribute to trauma-related psychological conditions (8). Oxytocin regulates social behavior and emotional functioning by acting on brain regions and neurotransmitter systems to reduce stress and fear, though abnormalities in oxytocin are linked to mental health conditions. By modulating the amygdala, prefrontal cortex, and neurotransmitter systems, oxytocin facilitates social behaviors and emotions by reducing stress and anxiety but dysfunction may contribute to psychiatric disorders (17).

#### 4.1.1 Oxytocin and fear

The oxytocin system may play a role in buffering the fear of cancer recurrence (FCR) that often plagues breast cancer patients, potentially enhancing psychological resilience. FCR, defined as “fear, worry, or concern about the recurrence or progression of cancer,” affects over 20% of cancer survivors, with women being more susceptible to it than men (90). Some studies have found that breast cancer patients with higher levels of psychological resilience are less likely to experience FCR (91). Oxytocin acts on various brain regions to regulate the fear response. The mesogenic oxytocin system in the hypothalamus is activated during fear learning.

In contrast, oxytocin in the central nucleus of the amygdala reduces contextual fear responses, and oxytocin receptor activation in the nucleus accumbens promotes suggestive fear (92). In an animal experiment, mice lacking oxytocin receptors in the forebrain after weaning exhibited reduced freezing behavior and abnormal fear learning (93). Injecting synthetic or selective oxytocin receptor agonists in the basolateral area suppresses the expression of contextually conditioned fear in rats (94).

#### 4.1.2 Oxytocin and exercise

Exercise can play a significant role in promoting resilience in breast cancer patients by enhancing the oxytocin system. Exercise has been shown to improve cellular bioenergetics, regulate cellular metabolism, and reduce the inflammatory response, thereby supporting and protecting the central nervous system (95). An active lifestyle can enhance an individual’s resistance to stress, thus promoting resilience. Studies in mouse models have shown that exercise can contribute to symptom relief in various neurological disorders such as Huntington’s chorea, Parkinson’s disease, and Alzheimer’s disease and can also reduce the risk of recurrence of colon and breast cancer (96).

A retrospective study has shown that moderate physical activity can enhance resilience in postoperative breast cancer patients, although the underlying biological mechanism remains unclear (97). In a study involving mice with breast cancer, exercise training increased oxytocin secretion and decreased the activity of the PI3K/AKT axes (Phosphatidylinositol 3-Kinase/Protein Kinase B axes) and ERK axes(Extracellular Signal-Regulated Kinase axes), inhibiting tumor cell proliferation (98). Hence, exercise may promote resilience in breast cancer patients by stimulating oxytocin secretion and altering tumor cell metabolism to reduce growth and metastasis.

#### 4.1.3 Oxytocin and tissue regeneration

Oxytocin has been found to have the potential to promote wound healing by reducing leukocyte infiltration in granulation tissue, decreasing inflammatory cytokine release, and promoting vascular remodeling and maturation. Such an effect may suggest an improvement in resilience. Moderate inflammatory responses are necessary for resilience in the face of stress or injury, whereas an excessive inflammatory response is detrimental to resilience (99). Hence, the oxytocin system may regulate extreme inflammatory responses by promoting wound healing, clearing damaged cells or tissues of the organism, and enhancing the ability to adapt to traumatic events, promoting resilience. However, an animal experiment using male SKH-1-h pure-hybrid hairless mice did not observe an increase in the rate of wound healing in mice injected with oxytocin compared to the control group (100). Therefore, further investigations are necessary to verify whether oxytocin can promote tissue regeneration.

#### 4.1.4 Oxytocin and pain adaptation

In breast cancer patients, persistent pain is a common treatment-related side effect, affecting more than 10% of patients (101). However, the oxytocin system may play a role in promoting pain adaptation and psychological resilience. Studies have shown that breast cancer patients with high resilience levels have lower pain interference levels (102). In particular, patients with higher psychological well-being and resilience may exhibit more excellent pain adaptation. Oxytocin may contribute to this effect, as evidenced by its analgesic properties in neonates following delivery. Animal experiments suggest that this may be due to oxytocin’s ability to reduce the GABA-evoked calcium response and depolarize GABA drive in trigeminal neurons, thus increasing the pain threshold of neonates (103).

### 4.2 Regulation of resilience in breast cancer patients by modulating neuroendocrine function

Oxytocin is a neuropeptide that bidirectionally communicates between the neuroendocrine and immune systems to regulate acute inflammatory responses and chronic immune surveillance, though aberrant oxytocin signaling suppresses and enhances cancer progression depending on the specific tumor context. Targeting the oxytocin-mediated interface between the nervous and immune systems may provide opportunities to therapeutically modulate inflammation and immunity in various diseases (9). Multidirectional oxytocin signaling provides delicate neuroendocrine control over immune homeostasis, though dysregulation can contribute to immunopathology, highlighting the oxytocinergic system as a potential therapeutic target (104).

#### 4.2.1 The oxytocin system and the hypothalamic-pituitary-adrenal axis

In response to environmental threats, the HPA axis is activated and primarily mediates the central stress response system (105). Breast cancer diagnosis, a disease with a high mortality rate, is a severe traumatic event that can be very stressful for women. This intense stress can modulate changes in multiple pro-inflammatory cytokines, including interleukin-6 (IL-6) and tumor necrosis factor-α (TNF-α), leading to the appearance of depressive symptoms, such as sadness, lethargy, and lack of pleasure, and even to depression (106, 107). C-reactive protein is a biomarker of inflammation that lacks specificity. According to a small clinical study, high concentrations of C-reactive protein in plasma were associated with decreased levels of resilience in breast cancer patients. In contrast, patients with high levels of resilience tended to have higher progesterone levels, suggesting that high inflammatory levels are linked to low resilience (108). Another review suggests that activating pro-inflammatory transcriptional control pathways increases susceptibility to depression (109). The review proposes that the HPA axis modulates inflammation by upregulating or suppressing it in response to stress. When the HPA axis is activated by cancer as a stressor, it increases glucocorticoid release, which blocks the inflammatory cascade initiated by pro-inflammatory transcription factors and pathways such as the NF-kB pathway, thus suppressing the inflammatory response. This mechanism allows for a level of inflammatory activity that does not surpass the individual’s tolerance level. However, continuous activation of the HPA axis may lead to the emergence of glucocorticoid tolerance, where immune cells become insensitive to glucocorticoids (110). This could be why cancer patients experience anxiety and depressive symptoms despite elevated glucocorticoid levels. In contrast, resilience may mitigate the effects of stress on inflammation-related depressive symptoms by improving sleep quality and enhancing physical activity (111).

In rats treated with glucocorticoids or exposed to stress, oxytocin has been shown to have a stimulatory effect on cell proliferation, which suggests that oxytocin may protect hippocampal sites from the adverse effects of elevated glucocorticoids (20). The oxytocin system and the HPA axis have been found to regulate each other mutually, and some researchers suggest that dysregulation of oxytocin and cortisol release levels could predict susceptibility to PTSD. When a stressor is encountered, the activity of the HPA axis is activated, and cortisol is produced in response. However, central oxytocin can inhibit the secretion of adrenocorticotropic hormone (ACTH), thus reducing the production and release of cortisol, which is involved in the neuroendocrine stress response (112). Following a traumatic event, individuals can fine-tune HPA axis activity by modulating multiple brain pathways, including the prefrontal cortex, amygdala, and PVN, thereby altering the level of glucocorticoid release from the adrenal cortex. Glucocorticoids regulate an individual’s physiological and psychological state in response to a traumatic or stressful event through various physiological pathways that affect neurological activity, immunity, and metabolism. When individuals show resilience by actively coping with stress or difficulties, glucocorticoid levels are better regulated (113).

In an animal experiment, the researchers observed that unpredictable maternal separation (UMS) of rats led to reduced time spent in the central area of an open field compared to the standard feeding group, indicating increased anxiety levels in these rats. In contrast, rats subjected to predictable maternal separation (PMS) showed lower anxiety levels than the standard feeding group. Oxytocin and OXTR mRNA levels were significantly higher in the medial prefrontal cortex (mPFC) of rats in the PMS group (114). These findings are consistent with another study, where mPFC produced anxiolytic effects by engaging the corticotropin-releasing hormone binding protein (CRHBP), enhancing the activity of male postsynaptic layer 2/3 pyramidal cells through antagonism of CRH (corticotropin-releasing hormone). In this pathway, mPFC regulates oxytocin response to CR (115), suggesting that blocking the oxytocin pathway from the paraventricular nucleus of the hypothalamus (PVN) to the mPFC could increase the risk of anxiety in rats.

However, an animal experiment revealed contradictory results when researchers administered oxytocin receptor antagonists into the third ventricle of adult Wistar rats. These rats exhibited significantly higher plasma ACTH concentrations than controls 2 days after the end of stress, which recovered to control levels by day 20. This suggests that endogenous brain oxytocin prolongs the duration of the response to stress in rats (116). It is noteworthy that oxytocin has also been shown to increase anxiety. Research has identified crfr2α as an essential regulator of anxiety. In a separate animal experiment, long-term oxytocin administration resulted in selective splicing of hypothalamic adrenocorticotropic hormone-releasing factor receptor 2α (Crfr2α) in rats, leading to anxiety-like behavior in rats (117). Therefore, further research is necessary to explore the relationship between oxytocin and the HPA axis more deeply.

#### 4.2.2 The oxytocin system and estrogen

The oxytocin system may promote resilience by increasing estrogen receptor (ER) expression in the brain to counteract anxiety behavior or decreasing ER expression in breast cancer tissue to reduce tumor load and improve symptoms in breast cancer patients. Increased endogenous estrogen levels and exposure to exogenous estrogens through hormone replacement therapy and oral contraceptives are critical high-risk factors for breast cancer. Estrogen metabolites and estrogen receptors play a role in all stages of the estrogen-promoting carcinogenic process (118). Hypothalamic oxytocin expression is demonstrated to be estrogen-dependent (119). There are also various associations between estrogen and its receptors and the oxytocin system. For instance, administering oxytocin to newborn female prairie voles increases ER mRNA expression in the hypothalamus and hippocampus (84). However, after intraperitoneal oxytocin administration to mammary carcinoma mice (MC4-L2), the mRNA expression of miR-195 and its associated signaling pathways, such as dephosphorylated Akt and ERK, the oxytocin receptor, and Bax genes, was significantly increased.

Atosiban reversed decreased mRNA expression of ERα, PI3K, NF-κB, cyclin D1, and Bcl-2 genes. Although commonly considered an oxytocin antagonist, some evidence indicates that atosiban may act as a biased agonist (120). The oxytocin receptor is a G protein-coupled receptor that interacts with Gq and Gi proteins. Typically oxytocin binds the receptor and activates Gq proteins, but atosiban may preferentially activate Gi proteins instead. By shifting signaling to Gi pathways, atosiban could inhibit adenylate cyclase and block oxytocin’s activation of Gq cascades, antagonizing oxytocin’s effects (121). Another study showed that oxytocin could downregulate ERα mRNA levels and reduce ERα protein expression in MCF-7 cells (122). These studies suggest a direct anti-estrogen-dependent mitogenic effect of oxytocin.

The oxytocin system regulates breast cancer patient’s physiological and pathological processes through NF-κB. Studies have shown that oxytocin can reduce tumor volume in a mouse model of breast cancer by downregulating NF-κB and upregulating miR-195 expression (120). Additionally, NF-κB activity is downregulated by increased expression of oxytocin receptors, leading to a decrease in the inflammatory response (123). Inflammatory and autoimmune pathologies are often associated with aberrant NF-κB activity, which the ER mediates the inhibition of at various levels. The interaction between these regulators may be harnessed to treat cancer (124, 125). Furthermore, nuclear NF-κB transcriptional machinery is disrupted in breast tumors resistant to SERM after estrogen treatment, potentially indicating that inhibition of NF-κB may be one of the pathways by which estrogen promotes apoptosis in breast tumor cells (126).

Endocrine therapy targeting ER+ is a necessary treatment for breast cancer, with evidence suggesting that estradiol acting in the brain distributing ER can modulate anxiety and depressive behavior (69). For instance, the administration of ER antagonists into the hippocampus of female rats resulted in increased anxiety behaviors (127). The distribution of oxytocin receptors is also linked to the ER. Specifically, compared to tissues with low oxytocin receptor expression, more ER-positive tissues have a diffuse distribution of oxytocin receptors (128). Estradiol has been shown to upregulate oxytocin receptor expression in breast malignancy tissues (MCF-7), while progesterone has the opposite effect (129). Moreover, oxytocin receptor gene expression was increased up to 8.6-fold in the corresponding tissues of ER-positive patients compared to those of ER-negative patients (72). The complex interactions between the oxytocin system and estrogen signaling pathways highlight oxytocin’s multifaceted roles in both breast cancer progression and patient resilience, underscoring the need for additional research to unravel the nuances of oxytocin signaling in cancer.

#### 4.2.3 The oxytocin system and 5-hydroxytryptamine

In addition to its anxiolytic effects, oxytocin has been shown to play a crucial role in social behavior, stress reduction, and interaction with the 5-Hydroxytryptamine (5-HT) system. The relationship between oxytocin and the 5-HT system has been studied extensively, with findings suggesting that oxytocin promotes resilience in breast cancer patients by increasing 5-HT activity (130). A recent study used Venus cDNA to investigate the role of oxytocin in the 5-HT system. The researchers observed that around 50% of tryptophan hydroxylase immunoreactive neurons in the nucleus accumbens were positive for Venus after placing the cDNA variant of yellow fluorescent protein into the regulatory region of the mouse encoding the OXT-R gene. In addition, the injection of oxytocin in the septum of mice increased the release of 5-HT in this region. However, this effect was blocked by 5-HT2A/2C receptor antagonists (131). These results highlight the intricate relationship between oxytocin and the 5-HT system and provide new insights into how oxytocin may promote resilience and reduce anxiety and depression in breast cancer patients.

#### 4.2.4 Oxytocin and AVP

The intricate relationship between AVP and oxytocin and their roles in breast cancer patients are emerging areas of increasing research interest. AVP is produced constitutively by the hypothalamus and primarily acts on renal collecting duct cells, while oxytocin, structurally similar to AVP, is involved in cardiovascular regulation (132). Studies have shown that low social support, reduced touch with loved ones, or behavioral stress can negatively impact vascular endothelial function, increase the rate of coronary atherosclerosis formation, and decrease oxytocin release (133). The balance between oxytocin and AVP release is also associated with altered mood behavior in individuals (134). Both oxytocin and AVP can act on oxytocin receptors in the lateral ventricles of mice to enhance social recognition (135).

Interestingly, various breast cancer cell lines, including MCF-7 and Skbr3, aberrantly express AVP and its receptor, which may produce anti-apoptotic effects (89). Therefore, the abnormal release of AVP from these breast cancer cells may, in conjunction with oxytocin, participate in the regulation of the cardiovascular system while also affecting the emotional behavior of individuals and playing a role in the regulation of resilience. Further research is needed to fully understand the relationship between oxytocin and AVP in breast cancer patients and their potential implications for treatment.

#### 4.2.5 The oxytocin system and thyroid function

Decreased oxytocin levels in the hypothalamic circulation may contribute to thyroid dysfunction and decreased psychological resilience in breast cancer patients. Some researchers suggest that alterations in estrogen levels, often in breast cancer patients, may lead to decreased IRAP activity and reduced oxytocin metabolism in the hypothalamus. As a result, circulating oxytocin levels increase, leading to decreased release of thyroid-stimulating hormone (TSH) from the pituitary gland and ultimately causing thyroid dysfunction (136). This thyroid dysfunction may result in psychological symptoms, such as psychomotor retardation, pleasure deficits, loss of libido, cognitive changes, and appetite disorders. These symptoms can lead to attention and executive impairment, fatigue, reduced quality of life, and increased risk of anxiety or depression (137, 138).

Moreover, breast cancer patients have a higher prevalence of thyroid peroxidase autoantibodies, likely due to the co-expression of thyroid peroxidase in both the thyroid and some breast tissues. Lactoperoxidase, structurally similar to thyroid peroxidase, is expressed in breast tumor cells, leading to immune responses to common thyroid/mammary antigens (139). Breast cancer patients who undergo chemotherapy, radiation, and immunotherapy may also damage the thyroid, further contributing to thyroid dysfunction. Thus, diminished oxytocin activity in the hypothalamic circulation may lead to thyroid dysfunction and psychological difficulties in breast cancer patients, resulting in decreased mental and physical health resilience. The complex interactions between the oxytocin system and estrogen signaling pathways highlight oxytocin’s multifaceted roles in both breast cancer progression and patient resilience, underscoring the need for additional research to unravel the nuances of oxytocin signaling in cancer.

#### 4.2.6 Balancing oxytocin signaling in breast cancer: implications for progression and resilience

**Figure 1:**
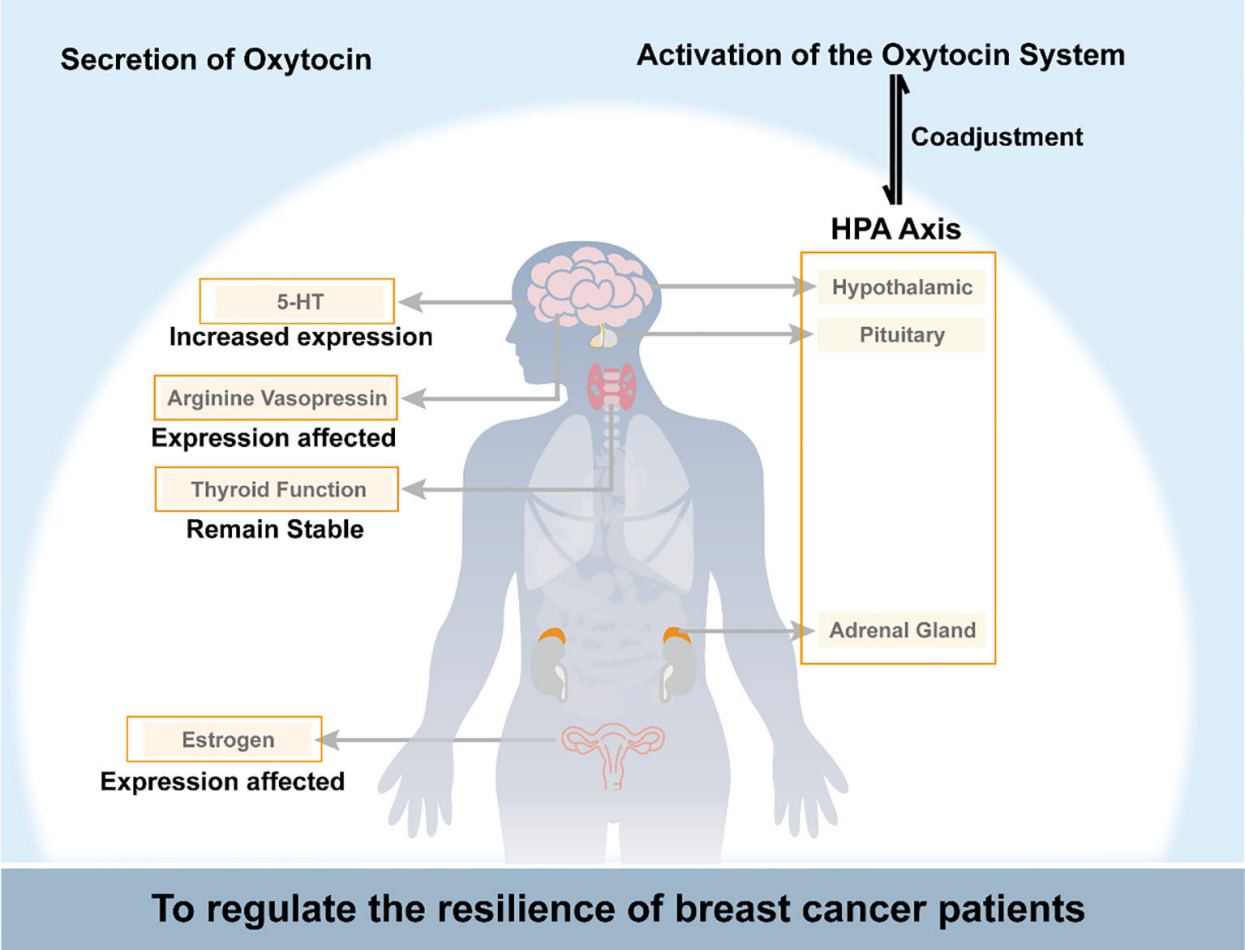
Schematic diagram of oxytocin regulating breast cancer patients’ psychological resilience by controlling the nervous system and endocrine function. HPA axis, the hypothalamic-pituitary-adrenal axis; 5-HT, 5-hydroxytryptamine.

Oxytocin interacts complexly with the HPA axis, estrogen signaling, the serotonin system, vasopressin, and thyroid function to impact breast cancer progression. It may counteract cancer-induced inflammation and glucocorticoid resistance by dampening HPA axis hyperactivity. However, prolonged oxytocin could also extend stress responses. Oxytocin exhibits anti-estrogenic properties by downregulating breast tumors’ estrogen receptors while activating estrogen receptors in healthy tissues. The balance between oxytocin and vasopressin signaling impacts mood and cardiovascular function in cancer patients. Decreased oxytocin may contribute to thyroid dysfunction and psychological symptoms. Overall, oxytocin plays multifaceted modulatory and compensatory roles, potentially inhibiting and promoting breast cancer depending on the physiological context. Further research is needed to elucidate the precise mechanisms by which oxytocin interfaces with other hormones to influence tumor development and patient resilience. As shown in **Figure 1**, oxytocin regulates breast cancer patients’ psychological resilience by controlling the nervous system and endocrine function.

## 5 Conclusion

**Figure 2:**
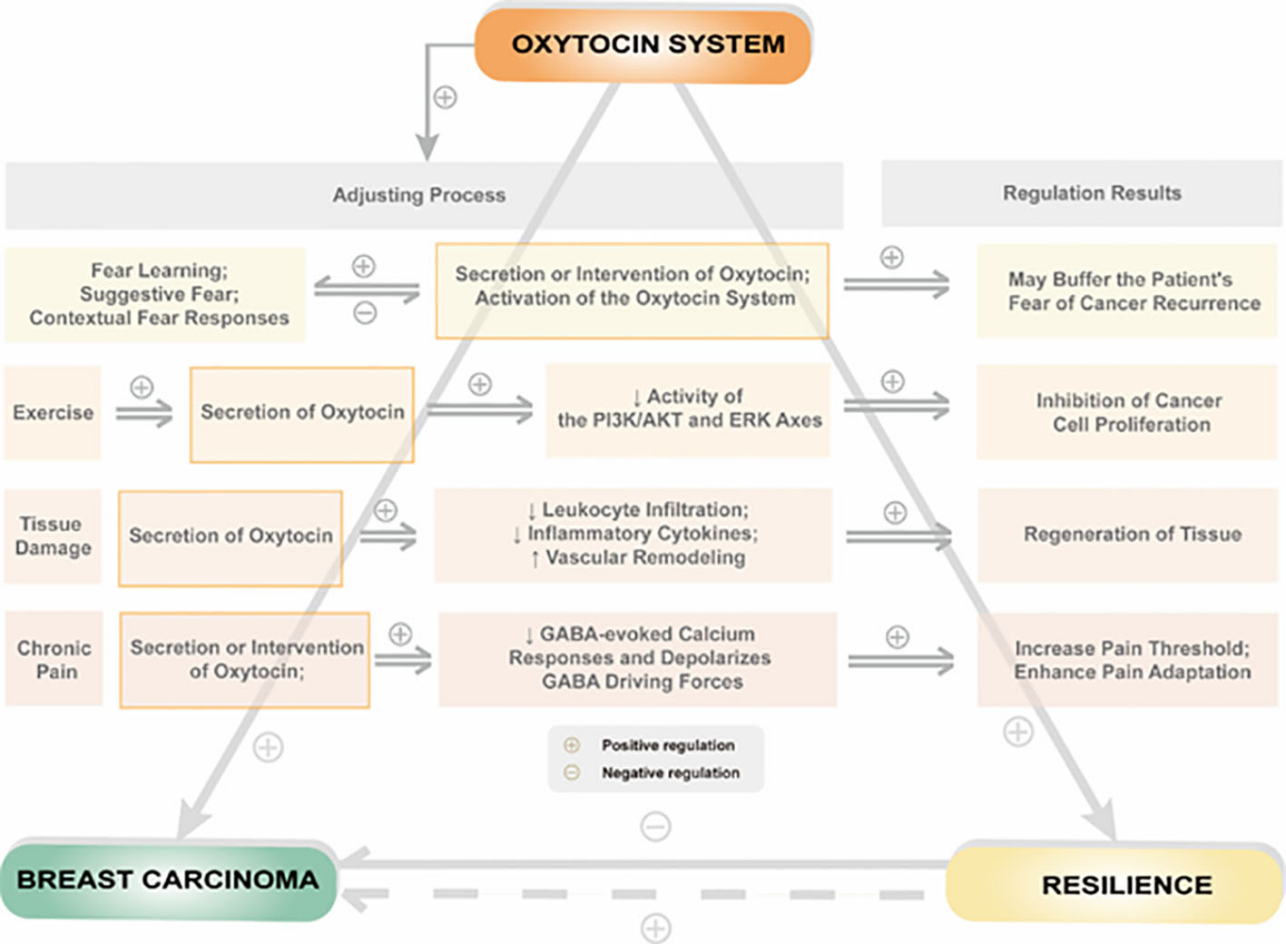
Relationship between psychological or physiological influences by regulation of the oxytocin system and resilience in patients with breast cancer.

Resilience formation and change mechanisms are complex and involve various biological and social factors. These mechanisms can be traced back to the evolution of mammals. The oxytocin system is a critical component of the neurobiological model of resilience and is involved in shaping resilience during fetal development and regulating resilience in response to social or traumatic events in adulthood. To illustrate the relationship between the oxytocin system and resilience, we propose a theoretical model shown in **Figure 2**.

Breast cancer is a traumatic event that can significantly affect a patient’s quality of life, and the level of resilience plays a critical role in how well a patient copes with the disease. Breast cancer patients often exhibit lower resilience levels than individuals without cancer, suggesting that the disease can negatively impact resilience. The oxytocin system regulates resilience in breast cancer patients in several ways. On the one hand, oxytocin acts directly on peripheral or central oxytocin receptors to regulate social behavior. On the other hand, oxytocin system activation can regulate psychological resilience by affecting estrogen and its receptors, the hypothalamic-pituitary-adrenal axis, thyroid function, metabolic levels of 5-HT, and the release of AVP in breast cancer patients. Additionally, oxytocin can indirectly affect resilience by influencing an individual’s ability to adapt to stressful situations, suppressing excessive inflammatory responses, and reducing pain.

Overall, the oxytocin system plays a significant role in regulating resilience in breast cancer patients. It is essential to consider the implications of these findings in clinical practice, particularly when diagnosing and treating breast cancer.

Currently, various measures are used to improve the resilience of breast cancer patients, mainly involving behavioral interventions or psychotherapy. Adjusting the release level of oxytocin may be the basis for the effectiveness of these interventions. In addition, moderate physical exercise also plays a vital role in improving resilience. In animal experiments, exercise can promote the release of oxytocin in mice, which may be one of the pathways through which exercise improves resilience. Intranasal administration of oxytocin has been shown to improve social communication deficits in disorders such as autism, which may be related to oxytocin’s direct action on central nervous system oxytocin receptors. In the future, plasma or salivary oxytocin levels and oxytocin genotypes could be used as predictive resilience indicators in breast cancer patients. Intranasal administration of oxytocin can be considered a pharmacological intervention pathway to improve the resilience of breast cancer women under the premise of strict adherence to safe doses.

Unfortunately, there is still no research on using oxytocin to improve resilience in breast cancer patients. Considering that oxytocin is involved in various functions of the female reproductive system and social behavior, oxytocin acting on the central nervous system is more likely to cause abnormal social behavior (140). Therefore, relevant research can be conducted, but the safe dosage of intranasal oxytocin administration for female patients must be determined.

## Author contributions

All authors proposed this manuscript’s concept, indicating a collective effort toward its development. To ensure the manuscript’s comprehensiveness, SL and RH conducted a comprehensive literature search. All authors collaborated in writing the first draft and continued to review and edit subsequent drafts until reaching a satisfactory final version. Finally, all authors participated in reading and approving the final version, which attests to the manuscript’s quality and accuracy. The dedication and collaboration of the authors highlight the importance of this topic, and their work will undoubtedly contribute to advancing the field’s knowledge on this matter. All authors contributed to the article and approved the submitted version.
